# Cas9-Mediated Gene Editing Using Receptor-Mediated Ovary Transduction of Cargo (ReMOT) Control in *Bombyx mori*

**DOI:** 10.3390/insects14120932

**Published:** 2023-12-07

**Authors:** Bin Yu, Sichen Dong, Xiaoyu Jiang, Liang Qiao, Jie Chen, Tian Li, Guoqing Pan, Zeyang Zhou, Chunfeng Li

**Affiliations:** 1 State Key Laboratory of Resource Insects, Southwest University, Chongqing 400715, China; yubin5868@outlook.com (B.Y.); dongsichen0017@163.com (S.D.); xiaojiang2021226@163.com (X.J.); jchen@swu.edu.cn (J.C.); lit@swu.edu.cn (T.L.); gqpan@swu.edu.cn (G.P.); 2 Chongqing Key Laboratory of Microsporidia Infection and Prevention, Southwest University, Chongqing 400715, China; 3 College of Life Sciences, Chongqing Normal University, Chongqing 401331, China; qiaoliangswu@163.com

**Keywords:** ReMOT control, *Bombyx mori*, lepidopteran insects, CRISPR/Cas9, gene editing

## Abstract

**Simple Summary:**

Lepidopteran insects are serious agricultural and forest pests that cause enormous global economic losses. The development of gene editing methods will significantly improve our ability to study gene function and greatly facilitate the control of lepidopteran pests. The application of gene editing technology in the model organism of lepidopteran insects, *Bombyx mori*, is mainly achieved by the microinjection of early embryos, but this approach is challenging in other lepidopteran insects. In this study, Cas9-mediated gene editing was established using the Receptor-Mediated Ovary Transduction of Cargo (ReMOT) control technique by injection into *B. mori* female pupae. We identified a *B. mori* oocytes-targeting peptide ligand (BmOTP) that mediated the transduction of Cas9 ribonucleoprotein into the oocytes, resulting in heritable gene editing of the offspring. Because the BmOTP ligand was highly conserved among lepidopteran, our results will significantly facilitate genetic manipulation of other lepidopteran insects, advancing pest control or economic insect breeding.

**Abstract:**

Lepidoptera is one of the most speciose insect orders, causing enormous damage to agricultural and forest crops. Although genome editing has been achieved in a few Lepidoptera for insect controls, most techniques are still limited. Here, by injecting female pupae of the Lepidoptera model species, *Bombyx mori*, gene editing was established using the Receptor-Mediated Ovary Transduction of Cargo (ReMOT) control technique. We identified a *B. mori* oocytes-targeting peptide ligand (BmOTP, a 29 aa of vitellogenin N-terminal of silkworms) with a highly conserved sequence in lepidopteran insects that could efficiently deliver mCherry into oocytes. When BmOTP was fused to CRISPR-associated protein 9 (Cas9) and the BmOTP-Cas9 ribonucleoprotein complex was injected into female pupae, heritable editing of the offspring was achieved in the silkworms. Compared with embryo microinjection, individual injection is more convenient and eliminates the challenge of injecting extremely small embryos. Our results will significantly facilitate the genetic manipulation of other lepidopteran insects, which is essential for advancing lepidopteran pest control.

## 1. Introduction

Lepidoptera, one of the most speciose insect orders [1], causes great economic damage to agricultural and forest crops. For instance, *Spodoptera frugiperda* (Lepidoptera: Noctuidae) has more than 350 host plants, such as corn and cotton [2]. It causes significant economic loss in the Americas and has recently invaded countries in Africa, Asia, and Oceania [3]. *Helicoverpa armigera* is a lepidopteran agricultural pest that causes severe damage to agricultural crops worldwide, such as cotton, tomato, and corn [4]. Hence, extensive research about the genomics and functional genomics is required to develop effective management strategies. To date, more than 30 genomic sequences of lepidopteran species have been obtained [1,5]. Genome editing has been achieved in a few lepidopteran insects. However, in most species, the editing techniques are still limited.

*Bombyx mori* (Lepidoptera: Bombycidae) is a model organism of Lepidoptera, and the gene-editing technology of silkworms has always been at the forefront of lepidopteran species. At present, zinc finger nuclease (ZFN) and transcription activator-like effector nuclease (TALEN) have been successfully applied in the targeted mutagenesis of silkworms using embryo injection [6,7]. As a simpler and more efficient gene-editing tool than ZFN and TALEN technologies, the CRISPR/Cas9 system has been widely used [8], and it also could mediate genome engineering in silkworms [9]. In Lepidoptera, CRISPR/Cas9 has been used in *S. frugiperda* [10], *S. litura* [11], *H. armigera* [12], *Agrotis ipsilon* [13], *Cydia pomonella* [14], *Papilio xuthus*, and *Vanessa cardui* [15,16]. However, most of these gene-editing tools rely on the microinjection of early embryos, which is difficult or inconvenient in other lepidopteran insects. Therefore, the development of gene-editing tools for a broader range of lepidopteran species is critical.

Recently, Receptor-Mediated Ovary Transduction of Cargo (ReMOT) control technology has become the alternative genome-editing strategy without embryo injections. In this method, an ovary-targeting peptide (a peptide of *Drosophila melanogaster* yolk protein, DmP2C) is fused with Cas9 (DmP2C-Cas9). After injecting the Cas9 ribonucleoprotein complex (Cas9 RNP; DmP2C-Cas9 protein complexed with guide RNAs) into the hemolymph of adult female insects, DmP2C transduces Cas9 RNP directly into the developing ovaries [17]. Using the DmP2C, ReMOT control has been shown to facilitate gene editing in many insect species, including *Aedes aegypti* [17], *Agrotis ipsilon* [13], *Anopheles stephensi* [18], *Anopheles sinensis* [19], *Culex pipiens pallens* [20], *Nasonia vitripennis* [21], *Tribolium castaneum* [22], *Diaphorina citri* [23], and even in the Chelicerate, *Ixodes scapularis* [24]. While the DmP2C ligand did not function robustly in *Bemisia tabaci*, an ovary-targeting peptide ligand of endogenous vitellogenin protein (BtKV) was identified to effectively mediate gene editing [25]. The ReMOT control overcomes the limitations of embryo injection by directly injecting the CRISPR/Cas9 system into the hemolymph of adult female insects. However, a gene-editing method based on the ReMOT control technique has not been established for Lepidoptera.

In this study, we developed a ReMOT control CRISPR-Cas9-based female pupae injection protocol for gene editing in *B. mori*. An oocytes-targeting peptide ligand (BmOTP) was identified, and injection of the Cas9 complex (Cas9 fused with BmOTP and complexed with 2 single-guide RNAs targeting to *BmBLOS2* [9]) into the hemolymph of vitellogenic females resulted in heritable editing of the offspring in silkworms without the need for embryonic microinjection.

## 2. Materials and Methods

### 2.1. Insect Rearing

The *B. mori* wild-type strain D9L was maintained in State Key Laboratory of Resource Insects at Southwest University (Chongqing, China) and reared on fresh mulberry leaves at 25 °C under a 12 h light/12 h dark photoperiod.

### 2.2. RNA Isolation and cDNA Synthesis

Three-day-old female pupae were grounded in liquid nitrogen, and the total RNA was extracted using a total RNA extraction kit (OMEGA, Norcross, GA, USA) according to the manufacturer’s instructions. After genomic DNA was digested with RNase-free DNase I (Takara, Tokyo, Japan) for 15 min at 37 °C, total RNA was used to reverse-transcribe the first-strand cDNA using a commercial kit (Yeasen, Shanghai, China). The cDNA samples were stored at −80 °C.

### 2.3. Plasmid Construction

The mCherry fragment was cloned by PCR, which was fused with an SV40 nuclear localization signal (NLS) and a (G_4_S)_2_ linker at the 5′ end, and a 3×FLAG and nucleoplasm NLS at the 3′ end. There were *Bam*H I (between the SV40 NLS and G4S linker) and *Hin*d III (between mCherry and FLAG) restriction enzyme sites. The mCherry fragment was inserted into the pET28a plasmid at *Nde* I and *Xho* I restriction enzyme sites to create the pET28-mCherry vector (Supplementary Sequences 1). The ovary-targeting ligands of silkworms (BmQV, BmVgN1, BmVgN2, BmVgN3, BmVgN2.1, and BmOTP) were cloned from the cDNA of female pupae by PCR. DmP2C was synthesized by Sangon Biotech (Shanghai, China). Then, the ovary-targeting ligands of silkworms and DmP2C were inserted into pET28-mCherry at the *Bam*H I restriction enzyme site using the One Step Cloning Kit (Yeasen, Shanghai, China). The sequences for expression of fusion proteins (the ovary-targeting ligands and mCherry) are listed in Supplementary Sequences 2. Cas9 was cloned by PCR with the G_4_S linker at the 5′ end. After digesting the pET28-BmOTP-mCherry with *Bam*H I and *Hin*d III to obtain the pET28-BmOTP fragment, the mCherry fragment was replaced with the Cas9 fragment to generate the pET28-BmOTP-Cas9 vector (Supplementary Sequences 3). All primers required for PCR reaction are listed in Table S1.

### 2.4. Protein Expression and Purification

All pET28 vectors were transformed into *E. coli Rosetta*. When the culture reached an OD600 of 0.4–0.6, the recombinant bacteria was induced with 0.1 mM isopropyl-β-D-thiogalactopyranoside for 20 h. The cells containing recombinant vector were resuspended in lysis buffer (20 mM Tris-HCl, pH 8.0, and 100 mM NaCl) and sonicated. Recombinant proteins were purified using the Ni–NTA beads according to the manufacturer’s instructions (QIAGEN, Hilden, Germany). Eluted proteins were dialyzed in dialysis buffer (50 mM Tris-HCl pH 8.0, 300 mM KCl, 0.1 mM EDTA, and 0.5 mM PMSF) using a dialysis bag (Sangon Biotech, Shanghai, China) at 4 °C, and the dialysis buffer was changed every 4 h, for a total of three times. Then, protein concentrations were estimated using Bradford Protein Assay Kit (Beyotime Biotechnology, Shanghai, China).

### 2.5. Guide RNA Generations

To knock out *BmBLOS2* gene in *B. mori*, two sgRNAs were used against *BmBLOS2* exon 2 and exon 4, following previous studies [9]. The sgRNAs were synthesized in vitro using a T7 high-yield RNA Transcription Kit (Thermo Fisher Scientific, Waltham, MA, USA) according to the manufacturer’s instructions. The primers required for PCR reaction are listed in Table S1.

### 2.6. Female Pupae Injection

To define the localization of fusion proteins in the embryo, female *B. mori* pupae were injected with 1 mg/mL mCherry fusion proteins (DmP2C-mCherry, BmQV-mCherry, BmVgN1-mCherry, BmVgN2-mCherry, BmVgN3-mCherry, BmVgN2.1-mCherry, and BmOTP-mCherry) once each on the 2nd and 4th days of the pupal stage, respectively, and a total of 20 μL was injected per silkworm each time. Recombinant mCherry fusion proteins lacking a targeting ligand and phosphate-buffered saline buffer (PBS) were injected as negative control. The mCherry fusion proteins or PBS injections were injected into female pupae through the stomata by a microliter syringe. On the 6th day of the pupal stage, ovaries were dissected, placed on a slide, and imaged using a fluorescence microscope (Olympus, Tokyo, Japan).

For gene editing, the 2 sgRNAs were mixed at a molar ratio of 1:1. BmOTP-Cas9 protein was mixed with the total sgRNAs (Table 1) and incubated at 25 °C for 20 min. Then Cas9 RNP was incubated with chloroquine, which was used as the endosome release reagent (ERR) [17]. The Cas9 RNP mix was injected into female pupae (20 μL per silkworm) once each on the 2nd and 4th days of pupal stage through the stomata by a microliter syringe. After injection, the pupae were reared at 25 °C until adult eclosion. The injected female moths (G_−1_) were crossed with the wild-type male moths, and G_0_ eggs were obtained.

### 2.7. Frozen Section of Ovarian Tissue

After injecting the mCherry fusion proteins into female pupae, the ovaries were dissected in PBS on the 6th day of pupal stage and washed with PBS. Then, an OCT embedding agent (Sakura, Torrance, CA, USA) was added, and the tissues were immersed in liquid nitrogen for embedding. The embedded ovarian tissue was sliced with a Cryotome (Thermo Fisher Scientific, Waltham, MA, USA), and the thickness was controlled at 40 μm. Tissue sections were immediately transferred to adhesive slides. Finally, the anti-fluorescence quenching agent (Beyotime Biotechnology, Shanghai, China) was added dropwise, and the sealing film was observed under a fluorescence microscope (Olympus, Tokyo, Japan).

### 2.8. ReMOT Control Mutation Analysis

After injection of G_−1_ female pupae, the G_−1_ female moths were crossed with wild-type males. The G_0_ progeny were screened for *BmBLOS2* gene-editing phenotypes (oily skin phenotype), which were easily evaluated at the larval stage. To identify oil mutations induced by the ReMOT control, the genomic DNA of oil silkworms were extracted using a Tissue DNA Kit (OMEGA, Norcross, GA, USA). Genomic DNA from the wild-type silkworm was used as a control. The DNA fragment spanning both T1 and T2 target sites was amplified with specific primers (Table S1) by PCR and cloned into pMD19-T for sequencing (Sangon Biotech, Shanghai, China).

### 2.9. Heritability Crosses

After injection of G_−1_ generation, the G_−1_ female moths were mated with wild-type males. G_0_ progenies were screened for the mutants. The G_0_ mutants were crossed with wild-type moths to demonstrate heritability of the mutant phenotype. For the G_0_ mosaic oily male silkworm, G_1_ oily female was crossed with wild-type male, and the G_2_ generation were self-crossed. For the G_0_ complete oily female, after the crossing of G_0_ mutant with wild-type male silkworm, the G_1_ generation were self-crossed to spawn G_2_ eggs.

## 3. Results

### 3.1. Identification of the BmOTP Ovary-Targeting Ligand

**Figure 1:**
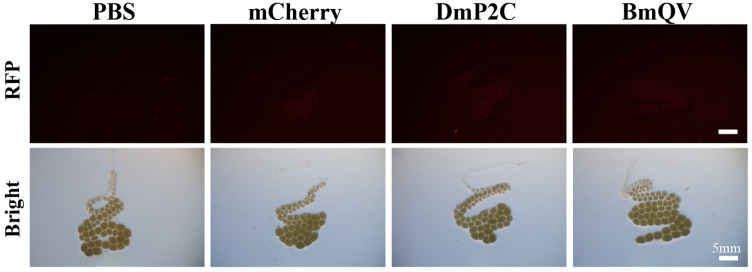
The delivery of cargo into silkworm ovaries. The DmP2C ligand from *D. melanogaster* and BmQV ligand (QGLFRKMETDV) from *B. mori* were fused with mCherry and injected into female pupae at days 2 and 4. Ovaries were dissected 2 days after injections and imaged using fluorescence microscope. PBS and mCherry were used as controls.

**Figure 2:**
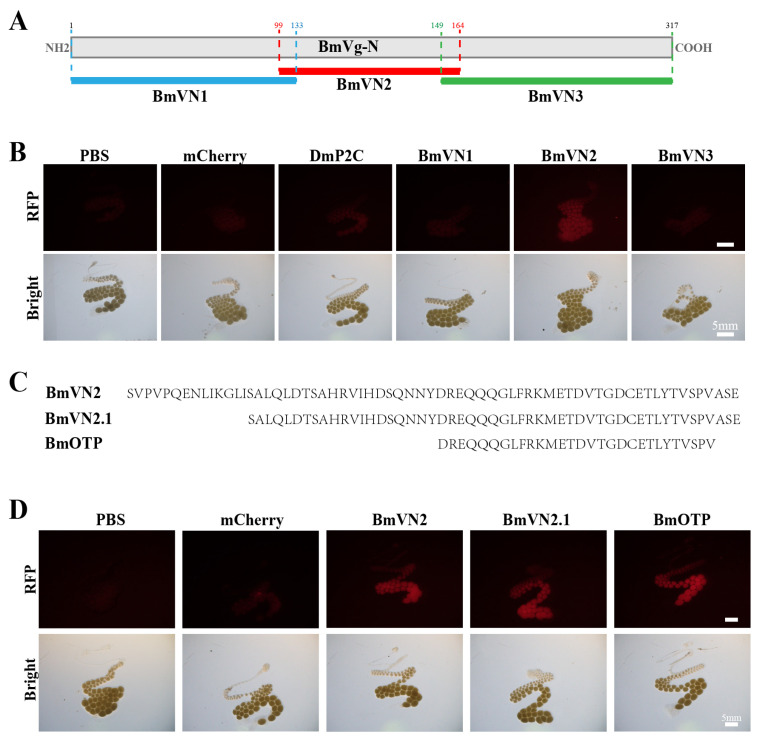
The deletion analysis of BmVg-mediated delivery of mCherry. (**A**): The schematic of BmVg-ligand segmentation. The 317 aa N-terminal portion of silkworm BmVg (BmVg-N) was divided into BmVgN1 (1–133), BmVgN2 (99–164), and BmVgN3 (149–317). The mCherry fused to these derivatives (BmVgN1, BmVgN2, and BmVgN3) to express the fusion proteins in *E. coli Rosetta* cells. (**B**): Targeting of mCherry by the BmVg ligands into the ovaries. The mCherry fusion proteins containing DmP2C or BmVg ligand fragments (BmVN1, BmVN2, and BmVN3) were injected into the hemolymph of silkworm female pupae. The ovaries were dissected and imaged using fluorescence microscope. PBS and mCherry were used as controls. (**C**): The sequences of BmVN2 region deletions. (**D**): Deletion analysis of the BmVN2 region. The mCherry fusion proteins containing BmVN2 ligand fragments (BmVN2, BmVN2.1, and BmOTP) were injected into female pupae and the ovaries were imaged. PBS and mCherry were used as controls.

The delivery of cargo into the silkworm ovaries was first tested with the DmP2C ligand. Our results demonstrated that there was no significant difference between DmP2C and the mCherry or PBS controls in the silkworms, indicating that DmP2C could not effectively enter the ovaries (Figure 1). To find an alternative ovary-targeting peptide ligand, *B. tabaci* predicted vitellogenin-A1-like was aligned with *B. mori* vitellogenin. The result showed that BtKV, a ligand-targeting cargo into the ovaries of *B. tabaci* [25], was highly similar to the N-terminal portion of silkworm vitellogenin protein (Figure S1). The conserved peptide ligand of the silkworm with *B. tabaci*, termed BmQV (QGLFRKMETDV), was fused with mCherry (BmQV-mCherry). Next, the recombinant BmQV-mCherry protein was injected into the female silkworms. No significant fluorescence signal was observed in the ovaries of the female pupae following BmQV-mCherry injection compared with the controls (Figure 1). After that, the 317 aa N-terminal portion of silkworm BmVg (BmVg-N), containing the conserved BmQV region, was divided into three segments (BmVgN1, BmVgN2, and BmVgN3) and fused with mCherry (Figure 2A). After injection of these fusion proteins, significant red fluorescence was observed in the developing oocytes from females injected with the BmVgN2-mCherry fusion protein, but not in the DmP2C-mCherry, BmVgN1-mCherry, and BmVgN3-mCherry-injected groups or the PBS- and mCherry-injected controls (Figure 2B).

**Figure 3:**
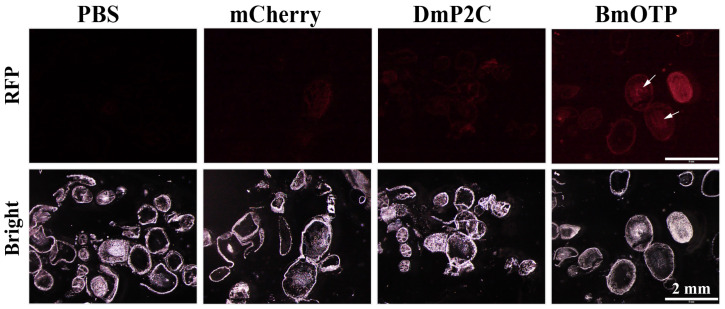
The frozen section analysis of BmOTP-mediated delivery into oocyte. Female pupae were injected with mCherry fused with DmP2C or BmOTP, and ovaries were dissected for frozen section. PBS and mCherry were used as injection controls. The frozen sections were imaged using a fluorescence microscope. The arrows showed mCherry protein entering the oocyte.

Deletion analysis was next utilized to determine if a smaller region of BmVgN2 was sufficient for uptake into the silkworm ovaries (Figure 2C). The results showed that a 29 aa fragment, termed BmOTP (DREQQQGLFRKMETDVTGDCETLYTVSPV), was identified as sufficient to deliver mCherry to the silkworm ovaries (Figure 2D). Frozen sections of ovarian tissue further confirmed that BmOTP efficiently directed cargo into the oocyte, whereas DmP2C did not (Figure 3). The alignment of vitellogenin N-terminal sequences in the Lepidoptera species showed that the BmOTP sequence was highly conserved (Figure S2), which suggested that the BmOTP ligand could deliver cargo into the ovaries of other lepidopteran species.

### 3.2. Gene Editing by ReMOT Control

**Figure 4:**
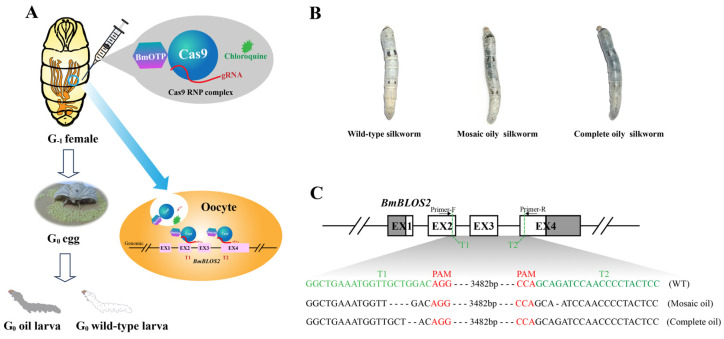
Cas9-mediated gene editing using ReMOT Control. (**A**): The flowchart of gene editing using ReMOT control in silkworm. The injection mixture was composed of BmOTP-Cas9 protein, sgRNAs (targeting exons 2 and 4 of *BmBLOS2* gene), and chloroquine (ERR) and injected into the hemolymph of G_−1_ female pupae. The mutant silkworm (oily phenotype) was screened in G_0_ offspring larva. (**B**): Mutants with oily skin induced by BmOTP-Cas9 RNP injection. After injection, there were 3 different phenotypes (wild-type, mosaic oily, and complete oily silkworms) in the G_0_ generation. (**C**): Molecular detection of G_0_ mutations. The targeting T1 sites at exons 2 (EX2) and T2 site at exons 4 (EX4) of sgRNAs are marked in green font. The protospacer adjacent motif (PAM) sites are red font. A small deletion was detected at the target region of *BmBLOS2* gene, causing the frameshift mutations.

Because the mutation of *BmBLOS2* resulted in an easily detectable oily skin phenotype [9,26,27], *BmBLOS2* was used as a target to confirm the gene editing by ReMOT control. Considering the expression level of BmVg reached the peak on the 3rd day of the pupal stage [28], G_−1_ female pupae were injected with Cas9 RNP complex (BmOTP-Cas9, sgRNAs targeting *BmBLOS2* exons 2 and exons 4, and ERR) once each on the 2nd and 4th day of the pupal stage, respectively, to achieve a higher transport efficiency and gene-editing efficiency (Figure 4A).

First, female pupae were injected with different concentrations of BmOTP-Cas9 to determine the optimal dose for gene editing (Table 1). No oily phenotype silkworm was observed as the pupae were treated with 0.1 μg/μL BmOTP-Cas9. When the protein concentration ranged from 0.5 to 2 μg/μL, there was a dose-dependent increase in *BmBLOS2* gene-editing efficiency. When the protein concentration exceeded 2 μg/μL, BmOTP-Cas9 precipitated from the solution and could not be used for injection.

The ERR is another element that requires optimization in the ReMOT control system. In this study, the chloroquine was used for ERR, and the survival rate of silkworms was examined after female pupae were injected with various doses of chloroquine. As showed in Figure S3A, the survival rate of the silkworms did not considerably decrease when using ≤20 mM chloroquine. But, it significantly decreased at concentrations >20 mM. The egg productions of silkworms injected with 15 mM and 20 mM chloroquine were also not significantly different from those of the control group (Figure S3B). The data showed that chloroquine concentrations ≤20 mM were tolerable. Silkworms with the oily phenotype were generated when female pupae were treated with 15 mM or 20 mM chloroquine, whereas no gene-editing events were observed when 10 mM chloroquine was used (Table 1).

After injection, two oily phenotypes with *BmBLOS2* mutations in G_0_ were observed: mosaic oily phenotype in male individuals and complete oily phenotype in female individuals (Figure 4B). To verify the knockout events of the oily phenotype, one mosaic oily silkworm and one complete oily silkworm were selected. Then, the silkworm genomic DNA was extracted after mating with wild-type (WT). The 4857 bp sequence containing the two target sites was amplified by PCR and sequenced. The sequencing results identified frameshift mutations due to base deletions in the oily silkworm but not in the wild-type (WT) silkworm (Figure 4C). These results suggested that deletions were common during ReMOT control in *B. mori*.

### 3.3. Heritability of Generated Mutations

**Figure 5:**
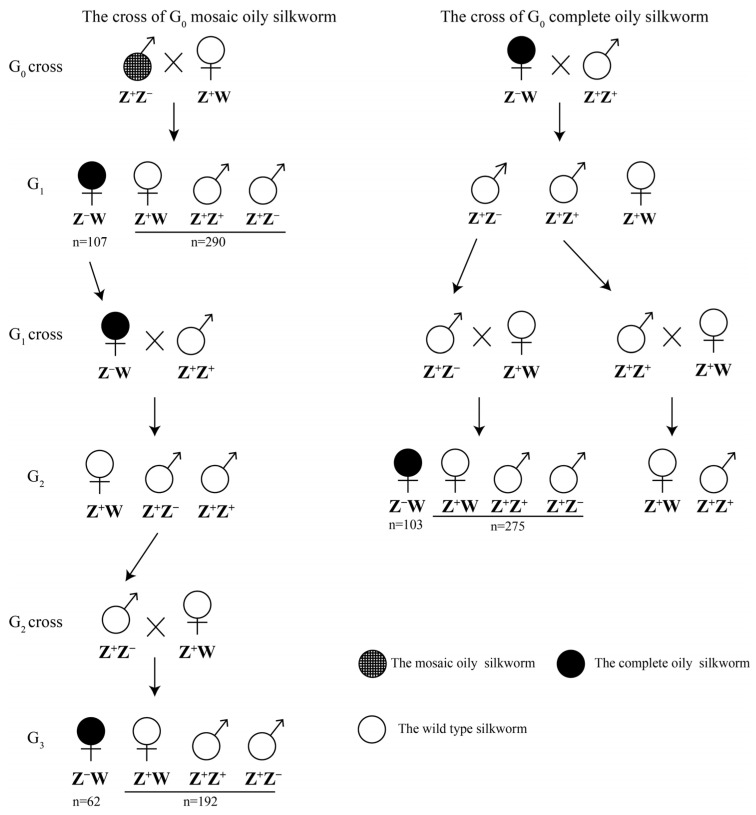
Crossing scheme to identify heritability. The mosaic oily male and complete oily female were screened in the G_0_ generation after the injection of G_−1_ females. For the G_0_ mosaic oily male silkworm, the G_0_ mutant male was crossed with wild-type female silkworm. G_1_ oily female was crossed with wild-type male, and the G_2_ generation were self-crossed. For the G_0_ complete oily female, after the crossing of G_0_ mutant with wild-type male silkworm, the G_1_ generation were self-crossed.

To demonstrate that the mutations generated by ReMOT control were heritable in *B. mori*, mutant silkworms were crossed with wild-type silkworms (Figure 5). *B. mori* uses a ZW/ZZ sex determination system, in which heterozygous ZW produces females and homozygous ZZ produces males. When the mosaic oily phenotype G_0_ male moth (ZZ) was crossed with the WT female moth (ZW), 107 G_1_ offspring had a complete oily phenotype and 290 G_1_ offspring showed a wild phenotype. All G_1_ offspring displaying a complete oily phenotype were female. The complete oily phenotype of G_1_ ZW silkworms disappeared in the G_2_ offspring after crossing with WT males. In the G_3_ offspring, 62 oily females and 192 WT offspring were detected (expected mutants account for 1/4).

When the complete oily phenotype G_0_ female silkworm (ZW) was crossed with the WT male moth (ZZ), the G_1_ offspring all exhibited a WT phenotype. After mating males in the G_1_ generation with WT females, 103 mutants and 275 wild-type G_2_ offspring were observed in the broods with mutants, and all mutant offspring were females (Figure 5). The results showed that the mutant phenotypes were heritable, and the germline was edited.

## 4. Discussion

In this study, we have developed a heritable CRISPR/Cas9 gene-editing system in *B. mori* using ReMOT control by switching the ovary-targeting ligand from DmP2C to BmOTP. DmP2C was derived from the yolk protein of *D. melanogaster* and used for the original ReMOT control method, which is effective for gene editing in a various of insect species, including mosquito [12,16,17,18], *N. vitripennis* [19], *T. castaneum* [20], and *D. citri* [23], as well as *I. scapularis* [24]. However, the DmP2C ligand failed to target the ovary in the silkworm. BtKV, an ovary-targeting peptide ligand of *B. tabaci*, can mediate efficient, heritable editing of the offspring genome [25] and was identified as highly similar to the N-terminal portion of silkworm vitellogenin protein (BmOTP) (Figure S1). In this study, BmOTP, a 29 aa ligand of silkworm vitellogenin protein, was identified to deliver the Cas9 RNP complex to silkworm oocytes, resulting in heritable gene editing of the progeny. The sequence of BmOTP was also highly conserved in Lepidoptera (Figure S2), suggesting that the BmOTP ligand could deliver Cas9 RNPs to the ovaries of other lepidopterans, providing a feasible and efficient option for lepidopteran insects that previously could not be genetically modified.

*B. mori* has been used as a model organism for gene-editing techniques in lepidopteran species, especially with the development of CRISPR/Cas technology. To date, silkworm gene editing has been chiefly accomplished by embryo injection, as initially described by Tamura et al. [29]. In contrast to embryo injection, which requires specialized equipment and highly-trained technicians, the ReMOT control system is convenient because it involves the direct injection of female pupae. In addition, diapause eggs exist in many silkworm strains, making embryo injection much more difficult. In comparison to the embryo injection, ReMOT control minimizes physical injury to the eggs by injecting during the pupal stage, making gene editing in the diapause strains of silkworms much easier.

In *B. mori*, the delivery of the CRISPR/Cas9 system based on plasmids was quick and convenient, with high stability in the silkworms [30,31,32,33]. However, Cas9- and sgRNA-positive individuals were only available in the G_1_ generation, and the gene-edited individuals were generated in the hybrid offspring of Cas9- and sgRNA-positive individuals. Even with the co-injection of Cas9 proteins and sgRNA in G_0_ eggs, the heritable gene-editing events can only be detected in the G_1_ generation. Moreover, the gene-editing phenotype in the G_0_ generation can also be identified when the CRISPR/Cas system is delivered to the embryo via mRNA [9] or protein [34]. Gene-editing events are randomized in the germ cells and/or somatic cells of G_0_ generation by embryo injection, and not all cells are affected, which results in some phenotypes in the G_0_ generation being completely non-heritable [35]. ReMOT control technology in silkworms allowed positive individuals to be screened for gene editing in the G_0_ generation (Figure 4), greatly reducing the time to obtain heritable offspring.

Notably, all complete oily G_0_ larvae were females, and all mosaic G_0_ larvae were males in this study. The complete oily G_0_ female larvae were homozygote (Z*^BLOS2−^*/W), according to the genetic analysis (Figure 5). Since fertilization occurs after egg laying in the domestic silkworm, the edited Z chromosome cannot be inherited in the G_0_ female offspring if the Cas9 RNP only works in the G_1_ pupal stage of injected females. Therefore, it is possible that the Z chromosome of the G_0_ male offspring was modified by undegraded Cas9 RNP in the fertilized eggs due to incomplete degradation of the Cas9 injected in the G_−1_ female pupa, indicating a longer editing window for ReMOT technology. In contrast to the mosaic G_0_ larvae generated by embryo injection [36], the oil phenotype was heritable in the mosaic larvae of the G_0_ males generated by ReMOT control. Approximately 1/4 of the females in the G_1_ generation were generated by mating the mosaic larvae of the G_0_ females with wild-type males. Therefore, we hypothesized that Cas9 RNP was not completely degraded after editing the *BmBLOS2* gene of the maternal Z chromosome in the oocyte, and the remaining Cas9 RNP then randomly edited the *BmBLOS2* gene of the paternal Z chromosome in the somatic cell, resulting in the *BmBLOS2* knockout chimeras with a mosaic oil phenotype of the G_0_ generation.

The efficacy of gene editing in this experiment is greatly underestimated since the *BmBLOS2* gene is located on the Z chromosome, resulting in the inability to screen for the Z^−^Z^+^ phenotype in males. Additionally, the use of ReMOT control technology is now mainly restricted to the production of knockouts. However, the spectrum of applications could be greatly expanded by utilizing delivery-modified Cas9 proteins or different base editors. Prime editing (PE) is a revolutionary genome-editing method that allows for the precise placement of all 12 nucleotide substitutions, short insertions, and short deletions using a catalytically impaired Cas9 fused to an engineered reverse transcriptase and a prime editing guide RNA [37], which is now widely used in many species [38,39,40,41]. The precise editing of subsequent individuals could be achieved in the future by delivering the PE system into the oocyte through ReMOT control. Recently, research has established an effective delivery of DNA to the ovary using ReMOT control based on the ability of GAL4 to bind to the UAS sequence [42]. Therefore, ReMOT control combined with these applications could quickly achieve gene knockout, knock-in, and expression control and has a wide range of applications, ultimately advancing lepidopteran pest control mechanisms.

## 5. Conclusions

The *B. mori* oocytes-targeting peptide ligand was identified in this work. Based on the BmOTP ligand, ReMOT control allows for CRISPR-Cas9 gene editing in *B. mori*. In contrast to embryo injection, the direct injection of female silkworm pupae is more convenient and makes gene editing in the diapause strains of silkworms much easier. Since the BmOTP ligand was highly conserved among lepidopteran insects, our results will also greatly facilitate the establishment of genetic manipulation in other lepidopteran insects, advancing pest control or economic insect breeding. Furthermore, this technology could be greatly expanded by utilizing the delivery of modified Cas9 proteins or different base editors.

## Figures and Tables

**Table 1:** Gene-editing efficiency by ReMOT control in *B. mori*.

BmOTP-Cas9 (μg/μL)	Total sgRNAs (μg/μL)	Chloroquine (mM)	No. of Females Injected (*n*)	No. of G_0_ Broods	No. of G_0_ Broods with Mutants	G_0_ Mutant Broods/G_0_ Broods (%)
0.1	0.05	20	35	33	0	0
0.5	0.25	20	55	51	1	1.96
1	0.5	20	55	49	1	2.04
2	1	20	30	26	1	3.85
2	1	15	20	16	2	12.5
2	1	10	20	18	0	0

## Data Availability

All the datasets in this study are available from the first author or corresponding author.

## Supplementary Materials

The following supporting information can be downloaded at https://www.mdpi.com/article/10.3390/insects14120932/s1, Figure S1: Identification of the BmVg ligand; Figure S2: Alignment of the amino acid sequences of vitellogenin N-terminal in Lepidoptera; Figure S3: The effect of chloroquine on silkworm; Table S1: Oligonucleotide primers; Supplementary Sequences 1: DNA sequences for expression of mCherry using the pET28a vector; Supplementary Sequences 2: DNA sequences for expression of fusion proteins (the ovary-targeting ligands and mCherry) using the pET28a vector; Supplementary Sequences 3: DNA sequences for expression of fusion protein BmOTP-Cas9 using the pET28a vector.
