# Supplementary material for: Cas9-Mediated Gene Editing Using Receptor-Mediated Ovary Transduction of Cargo (ReMOT) Control in Bombyx mori
Source: Insects. 2023 Dec 7;14(12):932. doi: 10.3390/insects14120932 (PMC10743513; doi:10.3390/insects14120932)
Supplement: Supplementary file 1 [file insects-14-00932-s001.zip › insects-2676694-supplementary.pdf]

Supplementary Materials

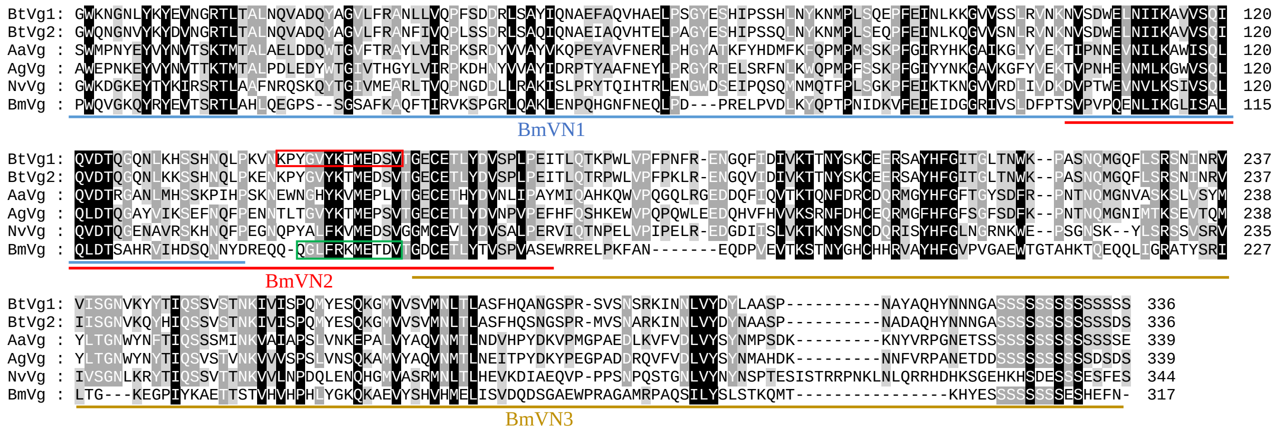

Figure S1. Identification of the BmVg ligand. *Bemisia tabaci* predicted vitellogenin-A1-like (BtVg1, XP_018912902.1), *B. tabaci* predicted vitellogenin (BtVg2, XP_018897090.1), *Aedes aegypti* vitellogenin (AaVg, AAA99486.1), *Anopheles gambiae* vitellogenin (AgVg, AAF82131.1), *Nasonia vitripennis* vitellogenin (NvVg XP_001607388.1) and *Bombyx mori* vitellogenin (BmVg, NP_001037309.1) were aligned using ClustalW in MEGA 8.0. The ''KV'' amino acids (red box), proven to deliver cargo into ovary of *B. tabaci*, were manually aligned to match the ''QV'' of the vitellogenin in *B. mori* (green box). The 317 aa N-terminal portion of silkworm BmVg (BmVg-N) was divided into three segments (BmVgN1, BmVgN2, and BmVgN3) and fused with mCherry to test the ability to deliver cargo into the oocyte of silkworm.

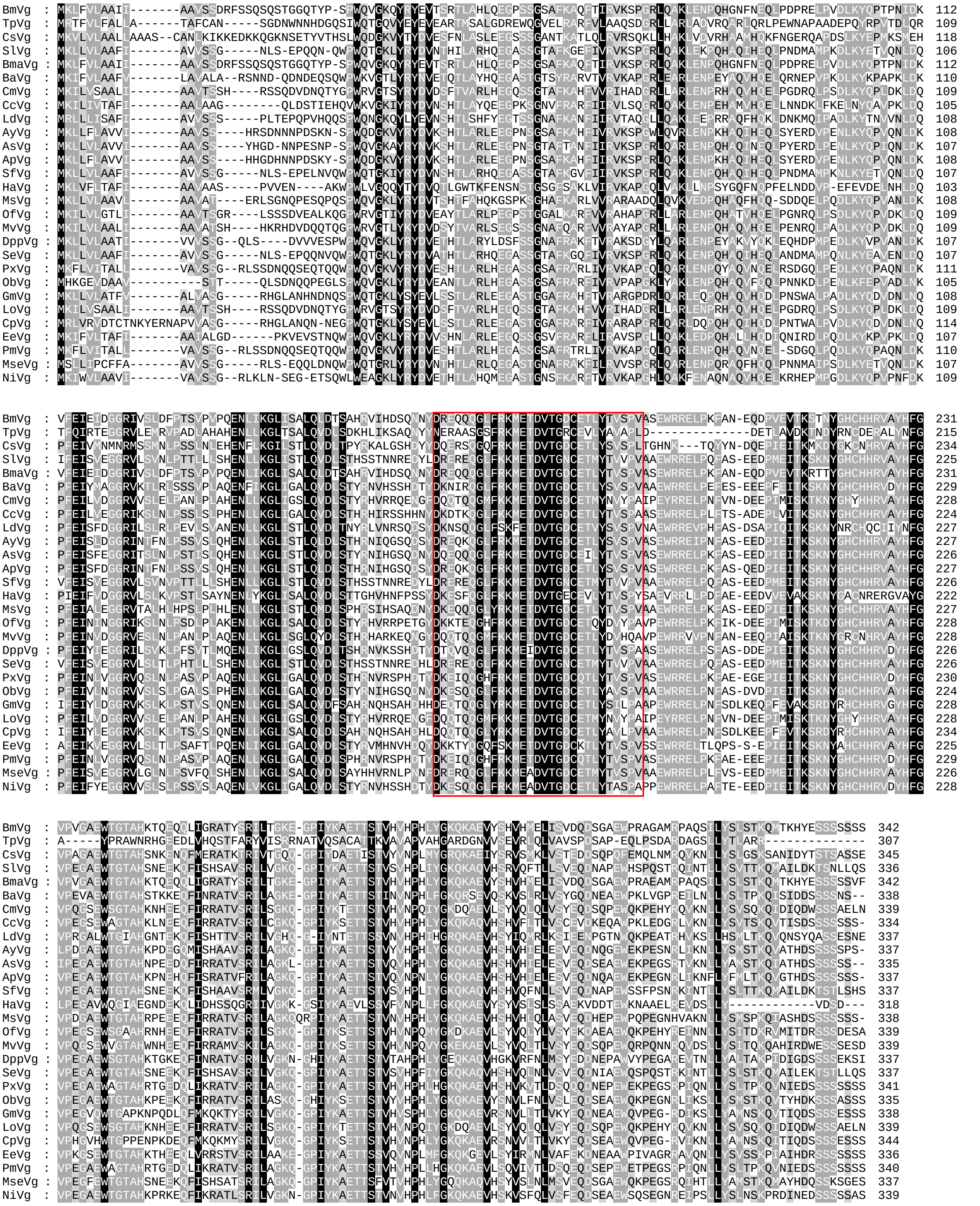

Figure S2. Alignment of the amino acid sequences of vitellogenin N-terminal in Lepidoptera. Multiple alignment was performed by using ClustalW in MEGA 8.0 software. Amino acid sequence alignment between *B. mori* (BmVg, NP_001037309.1), *Thitarodes pui* (TpVg, AWJ95280.1), *Conopomorpha sinensis* (CsVg, AXM43802.1), *Spodoptera litura* (SlVg, ABU68426.1), *Bombyx mandarina* (BmaVg, BAB32642.2), *Bicyclus*

anynana (BaVg, XP_023941468.1), *Cnaphalocrocis medinalis* (CmVg, AEM75020.1), *Corcyra cephalonica* (CcVg, AHZ89334.1), *Lymantria dispar* (LdVg, AAC02818.1), *Antheraea yamamai* (AyVg, BAF45319.1), *Actias selene* (AsVg, ABP63663.1), *Antheraea pernyi* (ApVg, BAB16412.1), *Spodoptera frugiperda* (SfVg, UAJ21054.1), *Helicoverpa armigera* (HaVg, XP_021195458.2), *Manduca sexta* (MsVg, XP_030021772.1), *Eumeta japonica* (OfVg, GBP26713.1), *Ostrinia furnacalis* (OfVg, QIH04838.1), *Maruca vitrata* (MvVg, AXY55008.1), *Danaus plexippus plexippus* (DppVg, OWR44310.1), *Spodoptera exigua* (SeVg, AOH73254.1), *Papilio xuthus* (PxVg, KPJ04900.1), *Operophtera brumata* (ObVg, KOB78233.1), *Grapholita molesta* (GmVg, WGM49048.1), *Leucinodes orbonalis* (LoVg, WGJ61535.1), *Cydia pomonella* (CpVg, WES10828.1), *Ephestia elutella* (EeVg, WDY35013.1), *Papilio machaon* (PmVg, XP_014366052.2), *Mythimna separata* (MseVg, AHG29547.1) and *Nymphalis io* (NiVg, XP_050356948.1). Identical and similar residues are highlighted in black and grey, respectively. The amino acids of BmOTP, proven to deliver cargo into ovary into *B. mori*, were aligned to match the conserved region of vitellogenin in Lepidoptera (red box).

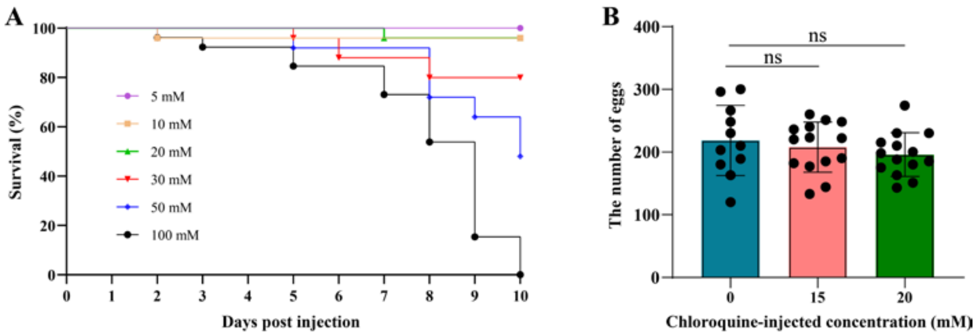

Figure S3. The effect of chloroquine on silkworm. A: The survival of chloroquine-injected females. At the 2nd day of pupal stage, different concentrations of chloroquine were injected into the hemolymph of females and the survival rate is calculated (n=30). B: The number of eggs was evaluated, after chloroquine-injected females pupae emerged and hybridized with wild-type male moths.

Table S1. Oligonucleotide primers

Primers	Primer sequences (5′-3′)
**For mCherry vector**	
>NLS-F	CCTGGTGCCGCGCGGCAGCCATATGCCAAAGAAGAAGCGGAAGGTCGGATCCGGTGGTG
>mCherry-F	AGGTCGGATCCGGTGGTGGAGGAAGCGGTGGAGGCGGGAGCATGGTGAGCAAGGGCGAG
>mCherry-R	CGTGGTCCTTATAGTCAAGCTTCTTGTACAGCTCGTCCATGCC
>FLAG-R1	GCTTGACTATAAGGACCACGACGGAGACTACAAGGATCATGATATTGATTACAAAGACG
>FLAG-R2	GCCGGCCTTTTTCGTGGCCGCCGGCCTTTTCTTATCGTCATCGTCTTTGTAATCAATAT
>NLS-R	TGGTGGTGGTGGTGGTGCTCGAGCTTTTTCTTTTTTGCCTGGCCGGCCTTTTTCGTGGC
>DmP2C-F	AAGAAGAAGCGGAAGGTCGGATCGAATCTGCAGCAGCAGCGC
>DmP2C-R	ACCGCTTCCTCCACCACCGGATCCGTTCTTAACCTCCTCGCT
>BmQV-F	AAGAAGAAGCGGAAGGTCGGATCGCAGGGACTTTTCAGGAAAATGGAAACTGATGTG
>BmQV-R	ACCGCTTCCTCCACCACCGGATCCCACATCAGTTTCCATTTTCCTGAAAAGTCCCTG
>BmVN1-F	AAGAAGAAGCGGAAGGTCGGATCGCCTTGGCAGGTTGGCAAAC
>BmVN1-R	ACCGCTTCCTCCACCACCGGATCCGTCGTAGTTGTTTTGAGAGT
>BmVN2-F	AAGAAGAAGCGGAAGGTCGGATCGTCAGTTCCTGTCCCCCAAG
>BmVN2-R	ACCGCTTCCTCCACCACCGGATCCTTCTGAAGCAACAGGAGAT
>BmVN3-F	AAGAAGAAGCGGAAGGTCGGATCGGGCGATTGTGAGACGCTGT
>BmVN3-R	ACCGCTTCCTCCACCACCGGATCCATTGAATTCGTGAGATTCCG
>BmVN2.1-F	AAGAAGAAGCGGAAGGTCGGATCGAGCGCTCTGCAACTTGACAC
>BmVN2.1-R	ACCGCTTCCTCCACCACCGGATCCTTCTGAAGCAACAGGAGAT
>BmOTP-F	AAGAAGAAGCGGAAGGTCGGATCGGACAGAGAACAGCAACAGGG
>BmOTP-R	ACCGCTTCCTCCACCACCGGATCCAACAGGAGATACCGTGTAC
**For Cas9 vector**	
Linker-F	TACACGGTATCTCCTGTTGGATCCGGTGGTGGAGGAAGCGGTGGAGGCGGGAGCGACAA
>Cas9-F	CGGTGGAGGCGGGAGCGACAAGAAGTACAGCATCGGCCT
>Cas9-R	TCCGTCGTGGTCCTTATAGTCAAGCTTGTCGCCTCCCAGCTGAGACAG
**For generating sgRNA targeting *BmBLOS2***	
> T1-F	AAGTGGCTGAAATGGTTGCTGGAC
> T1-R	TGCTGGACGTTTTAGAGCTAGAAA
> T2-F	AAGTGGAGTAGGGGTTGGATCTGC
> T2-R	GGAGTAGGGGTTGGATCTGCGTTT
>T7- T1-F	TATCGTGCTCTACAAGTGGCTGAAATGGTTGCTGGAC
>T7 -T2-F	TATCGTGCTCTACAAGTGGAGTAGGGGTTGGATCTGC
>gRNA-R	AACTTGAAAAAGTGGCACCGAGTCGGTGCTTTTTTTTGCGGC

For mutant analysis of *BmBLOS2*

>primer-F	GTAGGTAGTGTTTGAAGGGACA
>primer-R	CACGAGATAGCAGGGCACAA

Supplementary Sequences 1. DNA sequences for expression of mCherry using the pET28a vector. Color coding is as follows:

Magenta = His tag

Green = nuclear localization signal (NLS)

Orange = $(G_4S)_2$ linker

Red = mCherry

Purple = 3×FLAG tag

atgggcagcagccatcatcatcatcatcacagcagcggcctggtgccgcgcggcagccatatgccaaagaagaagcggaaggtcGGATCCggtggtggagg
aagcggtggaggcgggagcATGGTGAGCAAGGGCGAGGAGGATAACATGGCCATCATCAAGGAGTTCATGCGCT
TCAAGGTGCACATGGAGGGCTCCGTGAACGGCCACGAGTTCGAGATCGAGGGCGAGGGCGAGGGCC
GCCCCTACGAGGGCACCCAGACCGCCAAGCTGAAGGTGACCAAGGGTGGCCCCCTGCCCTTCGCCTG
GGACATCCTGTCCCCTCAGTTCATGTACGGCTCCAAGGCCTACGTGAAGCACCCCGCCGACATCCCCG
ACTACTTGAAGCTGTCCTTCCCCGAGGGCTTCAAGTGGGAGCGCGTGATGAACTTCGAGGACGGCGG
CGTGGTGACCGTGACCCAGGACTCCTCCCTGCAGGACGGCGAGTTCATCTACAAGGTGAAGCTGCGC
GGCACCAACTTCCCCTCCGACGGCCCCGTAATGCAGAAGAAGACCATGGGCTGGGAGGCCTCCTCCG
AGCGGATGTACCCCGAGGACGGCGCCCTGAAGGGCGAGATCAAGCAGAGGCTGAAGCTGAAGGACG
GCGGCCACTACGACGCTGAGGTCAAGACCACCTACAAGGCCAAGAAGCCCGTGCAGCTGCCCGGCGC
CTACAACGTCAACATCAAGTTGGACATCACCTCCCACAACGAGGACTACACCATCGTGGAACAGTACG
AACGCGCCGAGGGCCGCCACTCCACCGGCGGCATGGACGAGCTGTACAAGAAGCTTgactataaggaccacga
cggagactacaaggatcatgatattgattacaaagacgatgacgataagaaaaggccggcggccacgaaaaaggccggccaggcaaaaaagaaaaagctcgagc
accaccaccaccaccactga

Supplementary Sequences 2. DNA sequences for expression of fusion proteins (the ovary targeting ligands and mCherry) using the pET28a vector. Color coding is as follows:

Magenta = His tag

Green = nuclear localization signal (NLS)

Orange = $(G_4S)_2$ linker

Blue = the ovary targeting ligands

Red = mCherry

Purple = 3×FLAG tag

BmOTP-mCherry:

atgggcagcagccatcatcatcatcatcacagcagcggcctggtgccgcgcggcagccatatgccaaagaagaagcggaaggtcGGATCGGACAGAG
AACAGCAACAGGGACTTTTCAGGAAAATGGAAACTGATGTGACTGGCGATTGTGAGACGCTGTACAC
GGTATCTCCTGTTGGATCCggtggtggaggaagcggtggaggcgggagcATGGTGAGCAAGGGCGAGGAGGATAACAT
GGCCATCATCAAGGAGTTCATGCGCTTCAAGGTGCACATGGAGGGCTCCGTGAACGGCCACGAGTTCG
AGATCGAGGGCGAGGGCGAGGGCCGCCCCTACGAGGGCACCCAGACCGCCAAGCTGAAGGTGACCA
AGGGTGGCCCCCTGCCCTTCGCCTGGGACATCCTGTCCCCTCAGTTCATGTACGGCTCCAAGGCCTAC
GTGAAGCACCCCGCCGACATCCCCGACTACTTGAAGCTGTCCTTCCCCGAGGGCTTCAAGTGGGAGCG
CGTGATGAACTTCGAGGACGGCGGCGTGGTGACCGTGACCCAGGACTCCTCCCTGCAGGACGGCGAG
TTCATCTACAAGGTGAAGCTGCGCGGCACCAACTTCCCCTCCGACGGCCCCGTAATGCAGAAGAAGAC
CATGGGCTGGGAGGCCTCCTCCGAGCGGATGTACCCCGAGGACGGCGCCCTGAAGGGCGAGATCAAG
CAGAGGCTGAAGCTGAAGGACGGCGGCCACTACGACGCTGAGGTCAAGACCACCTACAAGGCCAAG
AAGCCCGTGCAGCTGCCCGGCGCCTACAACGTCAACATCAAGTTGGACATCACCTCCCACAACGAGG
ACTACACCATCGTGGAACAGTACGAACGCGCCGAGGGCCGCCACTCCACCGGCGGCATGGACGAGCT
GTACAAGAAGCTTgactataaggaccacgacggagactacaaggatcatgatattgattacaaagacgatgacgataagaaaaggccggcggccacga
aaaaggccggccaggcaaaaaagaaaaagctcgagcaccaccaccaccaccactga

BmQV:

CAGGGACTTTTCAGGAAAATGGAAACTGATGTG

BmVgN1:

CCTTGGCAGGTTGGCAAACAATACCGGTATGAGGTCACTTCTCGCACTTTGGCGCACTTGCAGGAGGG
TCCAAGCAGCGGAAGTGCTTTCAAAGCACAATTCACAATCCGGGTAAAGAGTCCTGGCCGACTCCAA
GCGAAACTAGAGAATCCACAGCATGGTAACTTCAATGAGCAACTCCCTGACCCGAGGGAACTGCCTGT
TGATCTCAAATACCAACCTACGCCGAACATCGATAAGGTATTCGAAATTGAAATTGACGGAGGTCGAAT
CGTAAGCCTCGATTTTCCAACTTCAGTTCCTGTCCCCCAAGAGAATTTAATAAAGGGTCTTATCAGCGC
TCTGCAACTTGACACGTCGGCTCACAGAGTCATTCACGACTCTCAAAACAACTACGAC

BmVgN2:

TCAGTTCCTGTCCCCCAAGAGAATTTAATAAAGGGTCTTATCAGCGCTCTGCAACTTGACACGTCGGCT
CACAGAGTCATTCACGACTCTCAAAACAACTACGACAGAGAACAGCAACAGGGACTTTTCAGGAAAA
TGGAAACTGATGTGACTGGCGATTGTGAGACGCTGTACACGGTATCTCCTGTTGCTTCAGAA

BmVgN3:

GGCGATTGTGAGACGCTGTACACGGTATCTCCTGTTGCTTCAGAATGGCGGCGAGAGCTTCCGAAGTT
CGCTAACGAACAGGACCCTGTTGAGGTTACAAAGAGCACAAATTACGGTCACTGTCACCACCGGGTC
GCTTATCACTTCGGAGTGCCGGTCGGTGCTGAGTGGACTGGAACAGCGCACAAAACCCAAGAACAAC
AGCTCATTGGCCGCGCTACTTACTCCCGTATCTTAACAGGCAAAGAGGGACCTATTTACAAAGCTGAAA
CCACGAGCACCGTTCATGTCCACCCTCATCTTTATGGTAAACAGAAAGCCGAAGTATACAGTCACGTCC
ATATGGAACTGATTTCAGTAGATCAAGACAGTGGGGCTGAATGGCCGAGAGCTGGAGCGATGCGTCCT
GCTCAAAGCATTTTATATTCCTTAAGCACAAAACAAATGACTAAGCATTACGAGTCTTCTTCGTCATCGT
CTTCTTCGGAATCTCACGAATTCAAT

BmVgN2.1:

AGCGCTCTGCAACTTGACACGTCGGCTCACAGAGTCATTCACGACTCTCAAAACAACTACGACAGAG
AACAGCAACAGGGACTTTTCAGGAAAATGGAAACTGATGTGACTGGCGATTGTGAGACGCTGTACAC
GGTATCTCCTGTTGCTTCAGAA

DmP2C:

AATCTGCAGCAGCAGCGCCAGCACGGCAAGAACGGCAACCAGGACTACCAGGATCAGAGCAACGAA
CAGAGGAAGAACCAGAGGACCAGCAGCGAGGAGGACTACAGCGAGGAGGTTAAGAAC

Supplementary Sequences 3. DNA sequences for expression of fusion protein BmOTP-Cas9 using the pET28a vector. Color coding is as follows:

Magenta = His tag

Green = nuclear localization signal (NLS)

Blue = the ovary targeting ligand BmOTP

Orange = $(G_4S)_2$ linker

Dark red = Cas9

Purple = 3×FLAG tag

atgggcagcagccatcatcatcatcatcacagcagcggcctggtgccgcgcggcagccatatgccaaagaagaagcggaaggtcGGATCGGACAGAG
AACAGCAACAGGGACTTTTCAGGAAAATGGAAACTGATGTGACTGGCGATTGTGAGACGCTGTACAC
GGTATCTCCTGTTGGATCCggtggtggaggaagcggtggaggcgggagcGACAAGAAGTACAGCATCGGCCTGGACAT
CGGCACCAACTCTGTGGGCTGGGCCGTGATCACCGACGAGTACAAGGTGCCCAGCAAGAAATTCAAG
GTGCTGGGCAACACCGACCGGCACAGCATCAAGAAGAACCTGATCGGAGCCCTGCTGTTCGACAGCG
GCGAAACAGCCGAGGCCACCCGGCTGAAGAGAACCGCCAGAAGAAGATACACCAGACGGAAGAACC
GGATCTGCTATCTGCAAGAGATCTTCAGCAACGAGATGGCCAAGGTGGACGACAGCTTCTTCCACAGA
CTGGAAGAGTCCTTCCTGGTGGAAGAGGATAAGAAGCACGAGCGGCACCCCATCTTCGGCAACATCG
TGGACGAGGTGGCCTACCACGAGAAGTACCCCACCATCTACCACCTGAGAAAGAAACTGGTGGACAG
CACCGACAAGGCCGACCTGCGGCTGATCTATCTGGCCCTGGCCCACATGATCAAGTTCCGGGGCCACT
TCCTGATCGAGGGCGACCTGAACCCCGACAACAGCGACGTGGACAAGCTGTTCATCCAGCTGGTGCA
GACCTACAACCAGCTGTTCGAGGAAAACCCCATCAACGCCAGCGGCGTGGACGCCAAGGCCATCCTG
TCTGCCAGACTGAGCAAGAGCAGACGGCTGGAAAATCTGATCGCCCAGCTGCCCGGCGAGAAGAAGA
ATGGCCTGTTCGGAAACCTGATTGCCCTGAGCCTGGGCCTGACCCCCAACTTCAAGAGCAACTTCGAC
CTGGCCGAGGATGCCAAACTGCAGCTGAGCAAGGACACCTACGACGACGACCTGGACAACCTGCTGG
CCCAGATCGGCGACCAGTACGCCGACCTGTTTCTGGCCGCCAAGAACCTGTCCGACGCCATCCTGCTG
AGCGACATCCTGAGAGTGAACACCGAGATCACCAAGGCCCCCCTGAGCGCCTCTATGATCAAGAGATA
CGACGAGCACCACCAGGACCTGACCCTGCTGAAAGCTCTCGTGCGGCAGCAGCTGCCTGAGAAGTAC
AAAGAGATTTTCTTCGACCAGAGCAAGAACGGCTACGCCGGCTACATTGACGGCGGAGCCAGCCAGG
AAGAGTTCTACAAGTTCATCAAGCCCATCCTGGAAAAGATGGACGGCACCGAGGAACTGCTCGTGAA
GCTGAACAGAGAGGACCTGCTGCGGAAGCAGCGGACCTTCGACAACGGCAGCATCCCCCACCAGATC
CACCTGGGAGAGCTGCACGCCATTCTGCGGCGGCAGGAAGATTTTTACCCATTCCTGAAGGACAACCG
GGAAAAGATCGAGAAGATCCTGACCTTCCGCATCCCCTACTACGTGGGCCCTCTGGCCAGGGGAAACA
GCAGATTCGCCTGGATGACCAGAAAGAGCGAGGAAACCATCACCCCCTGGAACTTCGAGGAAGTGGT
GGACAAGGGCGCTTCCGCCCAGAGCTTCATCGAGCGGATGACCAACTTCGATAAGAACCTGCCCAAC
GAGAAGGTGCTGCCCAAGCACAGCCTGCTGTACGAGTACTTCACCGTGTATAACGAGCTGACCAAAGT
GAAATACGTGACCGAGGGAATGAGAAAGCCCGCCTTCCTGAGCGGCGAGCAGAAAAAGGCCATCGTG
GACCTGCTGTTCAAGACCAACCGGAAAGTGACCGTGAAGCAGCTGAAAGAGGACTACTTCAAGAAA
ATCGAGTGCTTCGACTCCGTGGAAATCTCCGGCGTGGAAGATCGGTTCAACGCCTCCCTGGGCACATA
CCACGATCTGCTGAAAATTATCAAGGACAAGGACTTCCTGGACAATGAGGAAAACGAGGACATTCTGG
AAGATATCGTGCTGACCCTGACACTGTTTGAGGACAGAGAGATGATCGAGGAACGGCTGAAAACCTAT
GCCCACCTGTTCGACGACAAAGTGATGAAGCAGCTGAAGCGGCGGAGATACACCGGCTGGGGCAGGC
TGAGCCGGAAGCTGATCAACGGCATCCGGGACAAGCAGTCCGGCAAGACAATCCTGGATTTCCTGAA
GTCCGACGGCTTCGCCAACAGAAACTTCATGCAGCTGATCCACGACGACAGCCTGACCTTTAAAGAG
GACATCCAGAAAGCCCAGGTGTCCGGCCAGGGCGATAGCCTGCACGAGCACATTGCCAATCTGGCCG
GCAGCCCCGCCATTAAGAAGGGCATCCTGCAGACAGTGAAGGTGGTGGACGAGCTCGTGAAAGTGAT
GGGCCGGCACAAGCCCGAGAACATCGTGATCGAAATGGCCAGAGAGAACCAGACCACCCAGAAGGG
ACAGAAGAACAGCCGCGAGAGAATGAAGCGGATCGAAGAGGGCATCAAAGAGCTGGGCAGCCAGAT
CCTGAAAGAACACCCCGTGGAAAACACCCAGCTGCAGAACGAGAAGCTGTACCTGTACTACCTGCAG
AATGGGCGGGATATGTACGTGGACCAGGAACTGGACATCAACCGGCTGTCCGACTACGATGTGGACCA
TATCGTGCCTCAGAGCTTTCTGAAGGACGACTCCATCGACAACAAGGTGCTGACCAGAAGCGACAAG
AACCGGGGCAAGAGCGACAACGTGCCCTCCGAAGAGGTCGTGAAGAAGATGAAGAACTACTGGCGG
CAGCTGCTGAACGCCAAGCTGATTACCCAGAGAAAGTTCGACAATCTGACCAAGGCCGAGAGAGGCG
GCCTGAGCGAACTGGATAAGGCCGGCTTCATCAAGAGACAGCTGGTGGAAACCCGGCAGATCACAAA
GCACGTGGCACAGATCCTGGACTCCCGGATGAACACTAAGTACGACGAGAATGACAAGCTGATCCGG
GAAGTGAAAGTGATCACCCTGAAGTCCAAGCTGGTGTCCGATTTCCGGAAGGATTTCCAGTTTTACAA
AGTGCGCGAGATCAACAACTACCACCACGCCCACGACGCCTACCTGAACGCCGTCGTGGGAACCGCC

CTGATCAAAAAGTACCCTAAGCTGGAAAGCGAGTTCGTGTACGGCGACTACAAGGTGTACGACGTGC
GGAAGATGATCGCCAAGAGCGAGCAGGAAATCGGCAAGGCTACCGCCAAGTACTTCTTCTACAGCAA
CATCATGAACTTTTTCAAGACCGAGATTACCCTGGCCAACGGCGAGATCCGGAAGCGGCCTCTGATCG
AGACAAACGGCGAAACCGGGGAGATCGTGTGGGATAAGGGCCGGGATTTTGCCACCGTGCGGAAAGT
GCTGAGCATGCCCCAAGTGAATATCGTGAAAAAGACCGAGGTGCAGACAGGCGGCTTCAGCAAAGAG
TCTATCCTGCCCAAGAGGAACAGCGATAAGCTGATCGCCAGAAAGAAGGACTGGGACCCTAAGAAGT
ACGGCGGCTTCGACAGCCCCACCGTGGCCTATTCTGTGCTGGTGGTGGCCAAAGTGGAAAAGGGCAA
GTCCAAGAAACTGAAGAGTGTGAAAGAGCTGCTGGGGATCACCATCATGGAAAGAAGCAGCTTCGAG
AAGAATCCCATCGACTTTCTGGAAGCCAAGGGCTACAAAGAAGTGAAAAAGGACCTGATCATCAAGC
TGCCTAAGTACTCCCTGTTCGAGCTGGAAAACGGCCGGAAGAGAATGCTGGCCTCTGCCGGCGAACT
GCAGAAGGGAAACGAACTGGCCCTGCCCTCCAAATATGTGAACTTCCTGTACCTGGCCAGCCACTATG
AGAAGCTGAAGGGCTCCCCCGAGGATAATGAGCAGAAACAGCTGTTTGTGGAACAGCACAAGCACTA
CCTGGACGAGATCATCGAGCAGATCAGCGAGTTCTCCAAGAGAGTGATCCTGGCCGACGCTAATCTGG
ACAAAGTGCTGTCCGCCTACAACAAGCACCGGGATAAGCCCATCAGAGAGCAGGCCGAGAATATCATC
CACCTGTTTACCCTGACCAATCTGGGAGCCCCTGCCGCCTTCAAGTACTTTGACACCACCATCGACCG
GAAGAGGTACACCAGCACCAAAGAGGTGCTGGACGCCACCCTGATCCACCAGAGCATCACCGGCCTG
TACGAGACACGGATCGACCTGTCTCAGCTGGGAGGCGACAAGCTTgactataaggaccacgacggagactacaaggatca
tgatattgattacaaagacgatgacgataagaaaaggccggcggccacgaaaaaggccggccaggcaaaaaagaaaaagctcgagcaccaccaccaccaccact
ga
